# Parallel and non-parallel changes of the gut microbiota during trophic diversification in repeated young adaptive radiations of sympatric cichlid fish

**DOI:** 10.1186/s40168-020-00897-8

**Published:** 2020-10-29

**Authors:** Andreas Härer, Julián Torres-Dowdall, Sina J. Rometsch, Elizabeth Yohannes, Gonzalo Machado-Schiaffino, Axel Meyer

**Affiliations:** 1 Department of Biology, University of Konstanz, Universitätsstrasse 10, 78457 Konstanz, Germany; 2 Current address: Division of Biological Sciences, Section of Ecology, Behavior, & Evolution, University of California San Diego, La Jolla, California USA; 3 Current address: Department of Functional Biology, University of Oviedo, Oviedo, Spain

**Keywords:** *Amphilophus citrinellus*, Trophic ecology, Stable isotopes, Neotropical cichlids, Rapid adaptation, 16S rRNA gene sequencing, Parallel evolution, Nicaragua

## Abstract

**Background:**

Recent increases in understanding the ecological and evolutionary roles of microbial communities have underscored the importance of their hosts’ biology. Yet, little is known about gut microbiota dynamics during the early stages of ecological diversification and speciation. We sequenced the V4 region of the 16s rRNA gene to study the gut microbiota of Nicaraguan Midas cichlid fish (*Amphilophus* cf. *citrinellus*). Specifically, we tested the hypothesis that parallel divergence in trophic ecology in extremely young adaptive radiations from two crater lakes is associated with parallel changes of their gut microbiota.

**Results:**

Bacterial communities of fish guts and lake water were highly distinct, indicating that the gut microbiota is shaped by host-specific factors. Among individuals of the same crater lake, differentiation in trophic ecology was weakly associated with gut microbiota differentiation, suggesting that diet, to some extent, affects the gut microbiota. However, differences in trophic ecology were much more pronounced across than within species whereas similar patterns were not observed for taxonomic and functional differences of the gut microbiota. Across the two crater lakes, we could not detect conclusive evidence for parallel changes of the gut microbiota associated with trophic ecology.

**Conclusions:**

A lack of clearly differentiated niches during the early stages of ecological diversification might result in non-parallel changes of gut microbial communities, as observed in our study system as well as in other recently diverged fish species.

Video Abstract

## Background

The importance of microorganisms for many aspects of their hosts’ biology is increasingly recognized for a wide range of animals, from insects to mammals [1–3]. The gut microbiota is a complex and dynamic community that is fundamental for physiological processes, such as regulation of the immune system [4] and nutrient metabolism [5]. Further, the significance of microbes in animal evolution has become increasingly appreciated [1, 6, 7]. In some cases, the divergence of the gut microbiota appears to be strongly correlated with their host’s phylogeny and genetic divergence [8, 9]. These findings are supported by the fact that host genetics, together with environmental effects such as diet, contribute to shaping and maintaining gut microbiota composition [10–12]. However, open questions remain on how closely the gut microbiota matches the biology of its host during ecological diversification and speciation or whether the composition of the gut microbiota could even be predicted based on the ecology of its host. These important questions can best be addressed in a setting where evolution repeated itself, i.e., in pairs of species that evolved in parallel. Cases of parallel evolution of host species associated with divergence in trophic ecology and habitat use allow us to ask whether the gut microbiota also changes in a predictable and parallel manner. This question has been addressed in a few fish species covering a wide range of divergence times but results have been inconsistent. African cichlids from two old adaptive radiations of Barombi Mbo (0.5–1 myr) and Tanganyika (9–12 myr) show parallel changes of the gut microbiota associated with host diet [13]. Yet, studies on lineages that diverged more recently like whitefish and Trinidadian guppies did not find evidence for parallelism [14, 15], but see [16]. A recent study on Nicaraguan Midas cichlids (*Amphilophus* cf. *citrinellus*) found some evidence for an association between gut microbiota differentiation and the hosts’ phylogeographic history, but did not detect gut microbiota differentiation among sympatric species within the same crater lake [17].

**Fig. 1:**
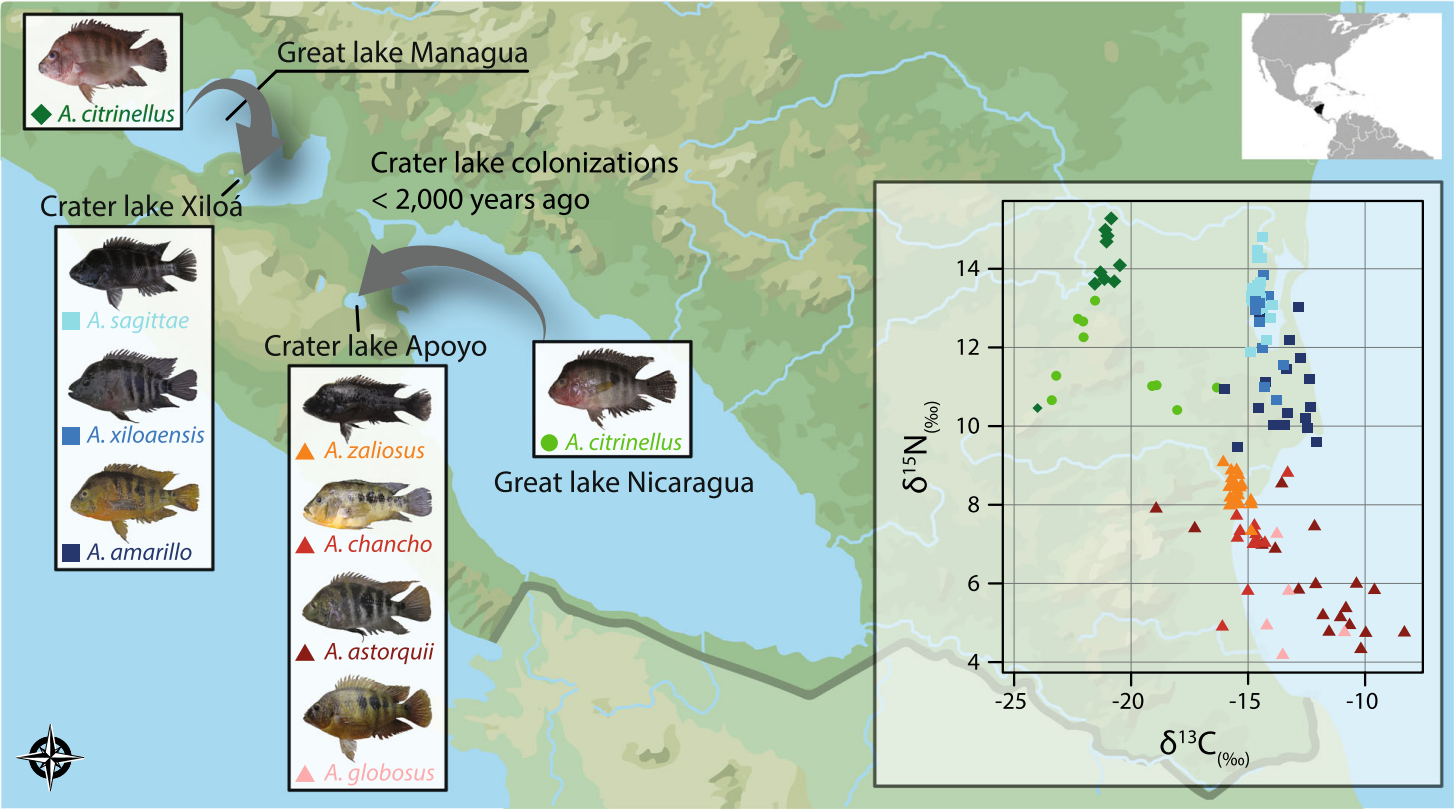
Map of Nicaragua showing the partial distribution of Midas cichlids in Nicaragua. Midas cichlids from the two great lakes (Managua and Nicaragua) colonized crater lakes Apoyo and Xiloá. In these two young crater lakes, multiple endemic species evolved in sympatry, representing a compelling case of parallel adaptive radiations. Within each crater lake, several deep-bodied, benthic and one elongated, limnetic species (*A. sagittae* in Xiloá and *A. zaliosus* in Apoyo) occur, which evolved rapidly within less than 2000 generations. White inset: Stable isotope analysis of carbon (δ^13^C) and nitrogen (δ^15^N) based on muscle tissue. δ^13^C and δ^15^N values vary significantly not only among environments, but also within each of the crater lakes where substantial variation can be observed among species. Within each of the crater lakes, variation in δ^15^N indicates differences in trophic ecology among species

Here, we focused particularly on the association between the gut microbiota and trophic ecology (measured by stable isotope ratios of carbon and nitrogen) of two very young adaptive radiations of Midas cichlids that evolved sympatrically and in parallel in two crater lakes. Specifically, we asked whether evolutionary divergence in host species’ trophic ecology can predict the composition of the gut microbiota. Currently, there are 13 described species of Midas cichlids [18–21] and their distribution results from independent colonization events from two older great lakes (Lakes Managua and Nicaragua) that are approximately 500,000 years old [19, 22] into several crater lakes that formed in calderas of inactive volcanoes (all crater lakes are between 1000 and 23,000 years old [23];). Crater lake Midas cichlids differ from their source populations of the great lakes in traits such as body shape and visual sensitivity [24–27]. The colonization events of crater lakes Apoyo (colonized from Lake Nicaragua) and Xiloá (colonized from Lake Managua) are estimated to have occurred as recently as 1700 and 1300 generations ago, respectively [20, 28]. Within these two crater lakes, multiple species of Midas cichlids evolved in sympatry during these extremely short time spans; hence, six and four species are endemic to Apoyo and Xiloá [20, 29]. Notably, one slender-bodied limnetic species (*A. zaliosus* in Apoyo and *A. sagittae* in Xiloá) independently evolved in each of the two crater lakes. These elongated limnetic species are not found in the great lakes and inhabit the open water zone that is exclusive to the deep crater lakes. Limnetic species differ distinctly in body shape from several deep-bodied benthic species in their respective crater lakes [20, 24, 27] and feed at a higher trophic level ([24]; Fig. 1). Previously, it has also been shown that gut microbiotas differ between a benthic-limnetic species pair from Apoyo [30].

The extraordinary system of crater lake Midas cichlids is a promising model to elucidate to what extent trophic ecology is mirrored by repeated and parallel changes of the gut microbiota in two very young adaptive radiations. Trophic ecology was determined by measuring stable isotope ratios of carbon and nitrogen, which provide information on different aspects of these fishes’ diet [31]. The isotope ratio of carbon (δ^13^C) indicates to what extent lacustrine organisms feed on benthic or limnetic food sources [32, 33], and the isotope ratio of nitrogen (δ^15^N) reflects this organism’s trophic position [34]. The composition of the gut microbiota was determined by sequencing the V4 region of the 16s rRNA gene. We investigated trophic ecology and the gut microbiota of Midas cichlids from the two source lakes, the great lakes Managua and Nicaragua, four species from crater lake Apoyo and three species from crater lake Xiloá (which represents a subset of Midas cichlid species endemic to these crater lakes). In particular, we tested the hypotheses that (i) species from distinct lakes differ in their gut microbiota but also from the bacterial communities of their natural environments (lake water) and (ii) repeated adaptation to different trophic niches is associated with parallel changes of the gut microbiota across the two crater lake radiations.

## Methods

### Sample collection

Specimens of the *Amphilophus* cf. *citrinellus* species complex were caught during field trips to Nicaragua in 2014 and 2015 (under MARENA permits DGPN/DB-IC-011-2014 & DGPN/DB-IC-015-2015). We collected *A. citrinellus* populations from the two great lakes, Lake Nicaragua (*n* = 10) and Lake Managua (*n* = 10). From the two crater lakes, we collected elongated limnetic species, *A. sagittae* from Xiloá (*n* = 19) and *A. zaliosus* from Apoyo (*n* = 19), as well as several deep-bodied benthic species, *A. amarillo* (*n* = 17) and *A. xilaoensis* (*n* = 20) from Xiloá, *A. astorquii* (*n* = 19), *A. chancho* (*n* = 10), and *A. globosus* (*n* = 6) from Apoyo (Fig. 1). All specimens were sacrificed by applying an overdose of MS-222 (400 mg/l). Then, whole guts were dissected, cleaned, and stored in absolute EtOH at − 20 °C until DNA extraction. Muscle tissue of the same specimens was collected in absolute EtOH and stored at − 20 °C for stable isotope analyses, as has been done in previous studies on this system [24]. Preservation methods can have an effect on stable isotope signatures, but ethanol preservation appears to only minimally affect carbon and nitrogen stable isotope signatures [35]. Further, we do not anticipate the preservation method to substantially influence our results since we performed a comparative study and we would not expect samples from different species to be differently affected. Four technical replicates of water samples were collected along the shores of the four lakes in 2018. Briefly, 500 ml of lake water was filtered through a cellulose nitrate filter (ø 25 mm, pore size 1 μm) for each replicate and filters were stored in Longmire’s solution [36] at − 20 °C until DNA extraction.

### Trophic analysis

Stable isotope ratios of carbon (δ^13^C) and nitrogen (δ^15^N) were determined based on muscle tissue of the same fish used for gut microbiota analyses. Dried and powdered samples (0.6 mg) were loaded into tin capsules and combusted in a vario Micro cube elemental analyzer (Elementar Analysensysteme, Germany). The resulting gases were fed via gas chromatography into the inlet of a Micromass Isoprime Isotope Ratio Mass Spectrometer (Isoprime, Cheadle Hulme, UK). Two sulfanilamides (Iso-prime internal standards) and two Casein standards were used. Internal laboratory standards indicated measurement errors (SD) of ± 0.03‰ for δ^13^C and 0.12‰ for δ^15^N. Isotopic values are reported in δ-notation in parts per thousand deviations (‰) relative to international standards for carbon (Pee Dee Belemnite, PDB) and nitrogen (atmospheric N2, AIR) according to the following equation:

1 $$ \delta\ \left({\mbox{\fontencoding{U}\fontfamily{wasy}\selectfont\char104}} \right)=1000\ast \left(\frac{R_{\mathrm{sample}}}{R_{\mathrm{standard}}}-1\right) $$

### Library preparation and Illumina sequencing

Approximately 50–100 mg of midgut tissue was dissected. As our goal was to capture resident bacteria associated with their hosts, guts were cut open using sterile scissors and gut samples were rinsed with EtOH and intestine contents were manually removed using sterile forceps to eliminate transient bacteria. Prior to DNA extraction, gut samples were air-dried to allow for EtOH evaporation. DNA was extracted using the commercial QIAamp DNA Stool Mini Kit according to the manufacturer’s protocol (Qiagen, Hilden, Germany). DNA from water samples was extracted from cellulose nitrate filters using a QIAGEN DNeasy Blood & Tissue kit. All DNA extractions and PCR amplifications were performed under sterile conditions in a laminar flow hood to minimize contamination risk. DNA concentrations were measured on a Qubit v2.0 Fluorometer (Thermo Fisher Scientific, Waltham, MA). For each round of extractions, one negative control of sterile H_2_O was included which in no case yielded detectable DNA concentrations. We performed two sequential PCRs (as recommended by Illumina), and after each one, the amplified product was purified with HighPrep^TM^ PCR beads (MagBio Genomics, Gaithersburg, MD). For the first PCR, we used the 515F and 806R primer with a universal 5’ tail, as indicated in the Illumina Nextera library preparation protocol, for DNA amplification of the V4 region of the 16S rRNA gene (292 bp). Briefly, 50 ng (fish guts) or 2 ng (water) of DNA were used as a template for the first PCR (2 min at 98 °C, 10 amplification cycles consisting of 15 s at 98 °C, 20 s at 55 °C and 20 s at 72 °C and a final elongation at 72 °C for 2 min) and the purified PCR amplicons were the template for the second PCR (2 min at 98 °C, 20 amplification cycles consisting of 15 s at 98 °C, 20 s at 67 °C and 20 s at 72 °C followed by a final elongation at 72 °C for 2 min) using primer including sequencing barcodes as well as the Illumina adapter sequences. Both PCRs were performed in 25-μl reaction volumes, amplifying with the Q5 High-Fidelity polymerase 2x Master Mix (New England Biolabs, Ipswich, MA). After purification, DNA concentrations were measured and specificity of amplification was checked for all samples using gel electrophoresis. Again, a negative control was included during each PCR but no amplified PCR products were detected (based on gel electrophoresis and measured DNA concentrations). Fish gut and water samples were separately pooled in an equimolar manner, and size selection was performed on a Pippin Prep device (Sage Science, Beverly, MA). The quality of the pooled libraries was assessed using a Bioanalyzer 2100 (Agilent Technologies, Waldbronn, Germany). Both libraries were paired-end sequenced, each in one lane of the Illumina flow cell. For the fish guts, we sequenced 2 × 250 bp on an Illumina HiSeq 2500 platform at TUCF Genomics (Tufts University, MA). Water samples were sequenced 2 × 150 bp on an Illumina HiSeq X-ten at the Beijing Genomics Institute (BGI, Hong Kong).

### Gut microbiota analysis

We obtained a total of 62,728,287 (median: 238,073 reads/specimen) and 111,949,556 (median: 6,825,739) raw sequencing reads that could be unambiguously assigned to a specific sample for fish guts and water samples, respectively. Illumina adapters were removed, and reads were trimmed with Trimmomatic v0.36 [37]. As there was no overlap between forward and reverse reads for water samples and the sequence quality of forward reads was consistently higher, we used 135 bp of the forward reads for all analyses. The demultiplexed and trimmed reads were imported into the open-source bioinformatics pipeline Quantitative Insights Into Microbial Ecology (QIIME2; [38]) to analyze microbial communities of fish guts and water samples. Briefly, sequence quality control was done with the QIIME2 plugin *deblur*. A phylogenetic tree of bacterial taxa was produced with FastTree 2.1.3 [39]. Different metrics of bacterial diversity (number of amplicon sequence variants (ASVs), Faith’s phylogenetic diversity and Shannon diversity) were calculated. For bacterial community composition, we calculated phylogenetic (weighted and unweighted UniFrac) and non-phylogenetic (Bray-Curtis dissimilarity) metrics [40, 41]. Weighted UniFrac takes into account the abundance of ASVs and, thus, can be strongly affected by highly abundant ASVs, especially if these are separated by long branches of the bacterial phylogeny. Unweighted UniFrac only takes into account presence or absence and therefore increases the impact of rare bacterial ASVs. We further included a non-phylogenetic metric (Bray-Curtis dissimilarity) as it might have a higher sensitivity to differences in bacterial community composition that are mainly driven by closely related bacterial ASVs [40, 41]. Taxonomy was assigned using vsearch [42] against the SILVA 132 ribosomal RNA (rRNA) databases at a 97% similarity threshold [43]. The weighted UniFrac distance matrix was visualized with principal coordinate analyses. Since most diversity metrics are sensitive to differences in numbers of reads per sample, we used 20,000 sequencing reads, the approximate number of reads for the sample with the fewest sequences, as our sampling depth for all further analyses. To determine whether this sampling depth was appropriate to capture a large proportion of microbial ASVs for each sample, we rarefied our data (Fig. S1). This analysis confirmed that a large proportion of microbial diversity is already captured at a sequencing depth of 20,000 reads/sample (Fig. S1). Non-parametric Wilcoxon rank-sum tests were used for pairwise comparisons [44] and Kruskal-Wallis tests for comparisons among multiple groups, as implemented in the R stats package [45]. To test for gut microbial community differences, both in terms of taxonomic and functional diversity, we applied Permutational Multivariate Analysis of Variance Distance Matrices (PERMANOVA; [46]), using the *adonis* function of the R vegan package. Correlations between distance matrices of the gut microbiota (weighted UniFrac, unweighted UniFrac, Bray-Curtis dissimilarity) and stable isotope data (δ^13^C or δ^15^N) were calculated using the *mantel.rtest* function of the R ade4 package [47]. Besides, correlations between pairwise distances of stable isotope data and gut microbiotas were also tested among individuals using Pearson’s product-moment correlation. MetaCyc pathway abundances were predicted based on 16S rRNA gene sequencing data with the PICRUSt2 plugin in QIIME2 [48]. As recommended by the developers, we calculated nearest-sequenced taxon index (NSTI) values (mean = 0.375, sd = 0.386) and used a maximum cutoff of 2 to exclude unreliable predictions based on poorly characterized bacterial taxa, which led to the exclusion of only 0.7% of sequence variants. Stable isotope ratios were normalized by *z*-score normalization to test for parallelism across crater lakes. We tested for effects of lake and stable isotope values on the abundance of bacterial taxonomic groups and MetaCyc pathways by using linear models (bacterial taxonomic group or Metacyc pathway ~ lake*normalized stable isotope ratio (δ^13^C or δ^15^N)). Bacterial taxonomic group and MetaCyc abundance was scored as parallel across crater lakes if stable isotope values had a significant effect (*P* < 0.05) on a given bacterial taxonomic group or MetaCyc and the interaction term between lake and stable isotope value was non-significant (*P* > 0.05). Only bacterial taxonomic groups with a mean proportional abundance of more than 0.1% were selected for the aforementioned analysis. Statistical analyses were performed in R v3.2.3 [49].

## Results

### Diet differentiation among Midas cichlids

In order to obtain information on trophic ecology of all studied Midas cichlid species, we measured stable isotope ratios of carbon (δ^13^C) and nitrogen (δ^15^N) which reflect littoral carbon usage and trophic level, respectively. Overall, Midas cichlids from different lakes significantly differed in δ^13^C (Kruskal-Wallis test, *P* < 0.001) and δ^15^N (*P* < 0.001; Fig. 1). Within each of the crater lakes, stable isotope ratios differed among species (δ^13^C: *P*_*Apoyo*_ < 0.001, *P*_*Xiloá*_ = 0.001; δ^15^N: *P* < 0.001 for both lakes). These results illustrate that sympatric species of crater lake Midas cichlids preferentially feed on different carbon sources and at different trophic levels based on nitrogen values, although some of the species overlap to a certain degree for both measures. Previous work on this system revealed that benthic and limnetic Midas cichlids mostly feed on similar diets but proportions of food items vary [24]. As predicted based on diet and inferred trophic niche [24, 29], the limnetic species had the highest nitrogen value in both crater lakes (Fig. 1). Benthic species occupied trophic niches that were generally at lower trophic levels (*A. globosus* in Apoyo, *A. amarillo* in Xiloá) than the limnetic species. Yet, one benthic species largely overlapped with the limnetic species in each crater lake (*A. chancho* with the limnetic *A. zaliosus* in Apoyo, *A. xiloaensis* with the limnetic *A. sagittae* in Xiloá). The benthic *A. astorquii* from Apoyo was highly variable in carbon and nitrogen signatures and largely overlapped with other species (Fig. 1).

### Gut microbiota differentiation across lakes

**Fig. 2:**
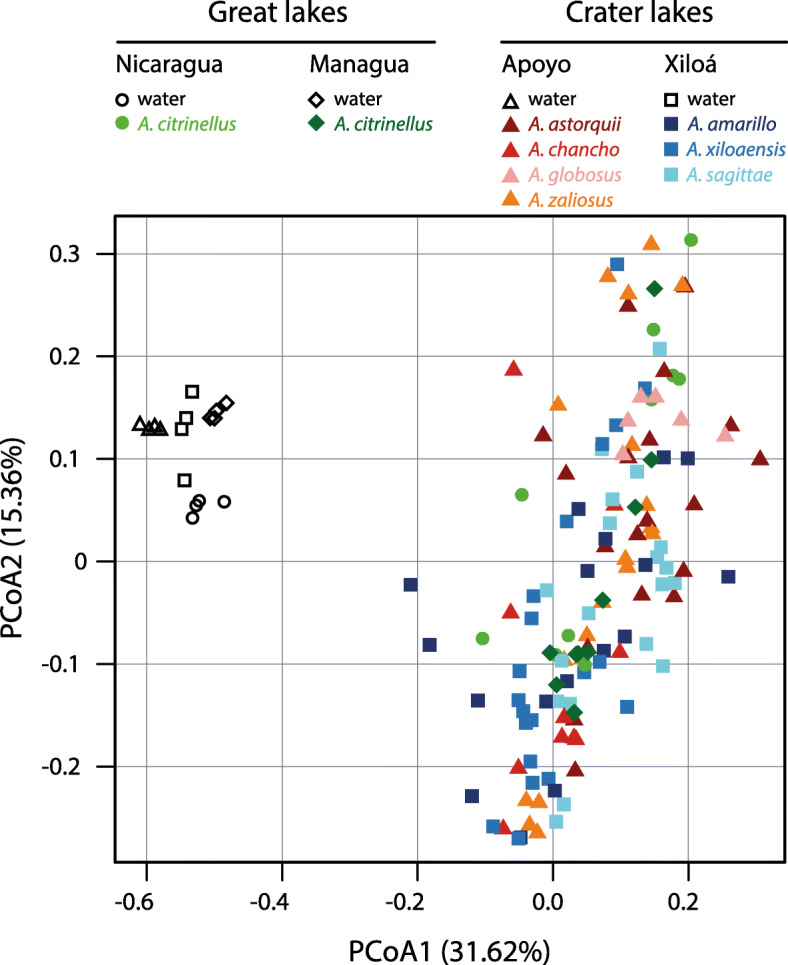
Principal coordinate analysis of bacterial community composition from Midas cichlids’ guts (colored symbols) and their natural environment (water, black symbols) measured as weighted UniFrac. Bacterial communities of the water samples are clearly differentiated from those of fish guts along PCoA1. This is confirmed by a PERMANOVA statistical test of bacterial community composition (adonis, *P* = 0.001). Among Midas cichlids, no apparent clustering by lake or species can be detected along PCoAs 1 and 2. Yet, PERMANOVA tests showed that bacterial community composition of fish guts differs among lakes (*P* = 0.001) and also between environment types (great lakes vs. crater lakes; *P* = 0.003)

**Fig. 3:**
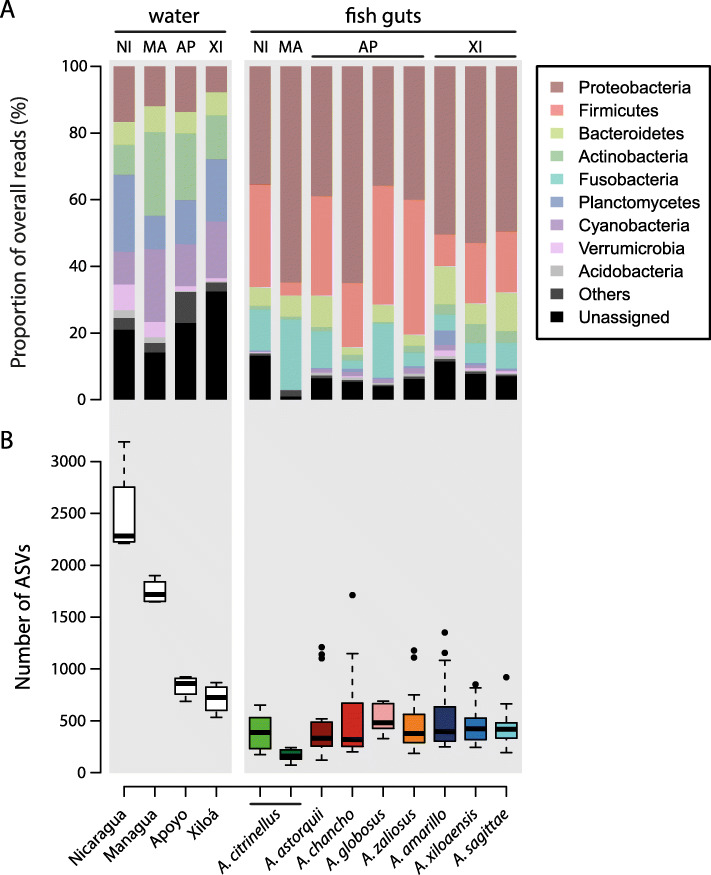
**a** Abundances of the nine most common bacterial phyla found in water and guts of the study species (> 0.5% of overall sequencing reads). The gut microbiota is dominated by Proteobacteria, Firmicutes, and Fusobacteria, three phyla which occur only at low abundance in the water. **b** Bacterial diversity (number of ASVs) is higher in the water compared to fish guts. Among Midas cichlid species, there is little variation, only *A. citrinellus* from Lake Managua shows remarkably reduced bacterial diversity

Bacterial community composition significantly differed between lake water and fish guts based on three metrics (weighted UniFrac, unweighted UniFrac, Bray-Curtis dissimilarity; adonis, *P* = 0.001 for all metrics; Fig. 2), emphasizing that the gut microbiota not merely represents the microbial community of the natural environment. In the water samples, *Cyanobacteria* (9.9–21.9%), *Planctomycetes* (10–23%), and *Actinobacteria* (9–25.1%) constituted a large proportion of microbial communities whereas these groups where much less abundant in the gut microbiota of Midas cichlids (Fig. 3a). In contrast, the gut microbiota was dominated by *Proteobacteria* (35.4–64.9%), *Firmicutes* (3.9–40.4%), and *Fusobacteria* (2.5–21.1%; Fig. 3a) whereas the last two where almost absent in the water. Bacterial diversity (number of ASVs, Faith’s phylogenetic diversity and Shannon diversity) was significantly higher in water (mean: 1446 ASVs) compared to fish guts (mean: 448 ASVs) (Wilcoxon rank-sum test, *P* < 0.001 for all metrics; Fig. 3a and Fig. S2). Great lakes showed a higher bacterial diversity than crater lakes for water samples (*P* < 0.001). Bacterial community composition of Midas cichlid guts differed not only across lakes (*P* = 0.001) but also between environment types (great lakes vs. crater lakes; *P* = 0.003) for all three metrics. Bacterial diversity was lower in great lake Midas cichlids (*P* < 0.001 for all metrics); however, this pattern disappeared when *A. citrinellus* from Lake Managua was removed from the analysis (*P* > 0.05 for all metrics). These results clearly show that bacterial diversity is largely constant in Midas cichlids from different environments, except for *A. citrinellus* from Lake Managua that showed strongly reduced bacterial diversity. Next, we investigated whether gut microbiota divergence within crater lakes is associated with the observed differences in stable isotope ratios.

### Association between trophic ecology and gut microbiota in crater lake adaptive radiations

The two crater lakes are each inhabited by several endemic Midas cichlid species that substantially differ in their morphology, ecological niche, and diet (Fig. 1). Yet, there were no significant differences in bacterial diversity (number of ASVs, Faith’s phylogenetic diversity and Shannon diversity) among sympatric species within each of the crater lakes (Wilcoxon rank-sum tests, *P* > 0.05 for all pairwise comparisons after Bonferroni correction). Hence, we tested whether bacterial community composition varied among species within each of the two parallel crater lake radiations of Apoyo and Xiloá (see Fig. S3 for weighted UniFrac data). There was overall differentiation in the taxonomic composition of bacterial communities among sympatric species in both crater lakes (*P*_*Apoyo*_ < 0.002, *P*_*Xiloá*_ < 0.01 for all three metrics). From the taxonomic composition of the gut microbiota, one can infer the functional bacterial metagenome by predicting the abundance of genes involved in metabolic pathways [48]. It should be noted that this method has limitations when studying wild-caught organisms whose microbes have not been thoroughly characterized. Yet, NSTI values were mostly low, suggesting that predictions of metabolic pathways are reliable for the gut microbial communities of Midas cichlids. Predicted functional bacterial metagenomes significantly differed among species only in crater lake Xiloá (*P* = 0.006).

**Fig. 4:**
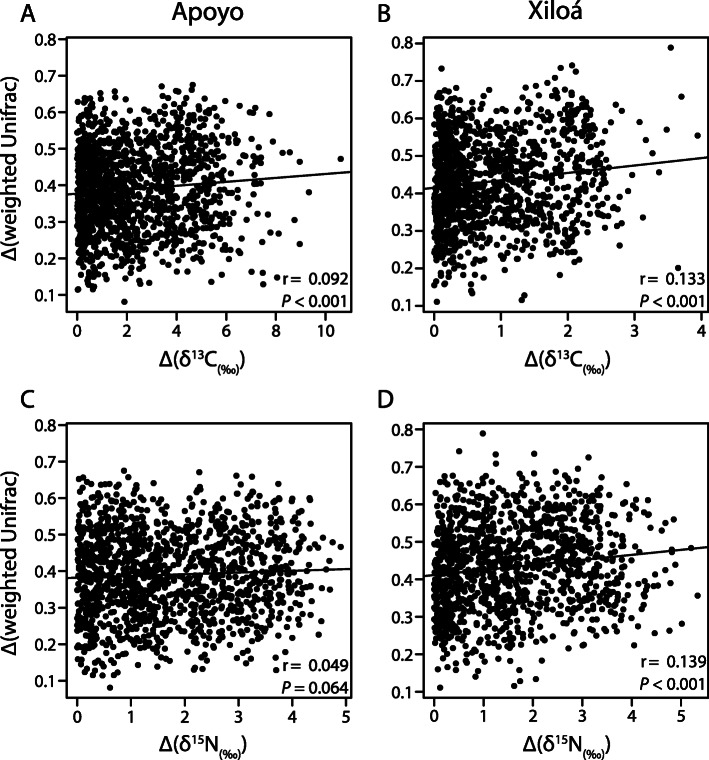
Pairwise distances of gut microbiota composition, Δ(weighted UniFrac), and trophic ecology, Δ(δ^15^N) and Δ(δ^13^C), among all individuals within crater lakes Apoyo (A&C) and Xiloá (B&D). Gut microbiota differentiation is positively correlated with divergence in carbon values in both crater lakes and with nitrogen values in crater lake Xiloá (Pearson’s product-moment correlation)

Since there is a pronounced variation in trophic ecology in both adaptive radiations (Fig. 1), we tested for correlations between the taxonomic composition of the gut microbiota with trophic ecology (δ^15^N and δ^13^C scores) using Mantel tests. We only found one significant correlation between weighted UniFrac and nitrogen signature in Xiloá (*r* = 0.133, *P* = 0.023) and suggested correlations between weighted UniFrac and carbon signature in Apoyo (*r* = 0.078, *P* = 0.092) as well as between unweighted UniFrac and carbon signature in Xiloá (*r* = 0.104, *P* = 0.098). Nonetheless, the proportion of variance explained by trophic ecology was relatively low in all cases. Further, we calculated pairwise distances of trophic ecology (δ^15^N and δ^13^C scores) and correlated these with pairwise distances in bacterial community composition among all individuals within each crater lake (Fig. 4). For carbon, we found a significant positive (Pearson’s product-moment correlation; weighted UniFrac: *r* = 0.092, *P* < 0.001), negative (unweighted UniFrac: *r* = − 0.082, *P* = 0.002) or no correlation (Bray-Curtis: *r* = − 0.008, *P* = 0.768) with bacterial community composition in Apoyo (Fig. 4a) and a significant positive correlation in Xiloá (weighted UniFrac: *r* = 0.133, *P* < 0.001; unweighted UniFrac: *r* = 0.141, *P* < 0.001; Bray-Curtis: *r* = 0.078, *P* = 0.028; Fig. 4b). For nitrogen, there was a significant positive (Bray-Curtis, *r* = 0.056, *P* = 0.038), suggested (weighted UniFrac: *r* = 0.049, *P* = 0.064) or no correlation (unweighted UniFrac: *r* = − 0.007, *P* = 0.783) in Apoyo (Fig. 4c) and a significant (weighted UniFrac: *r* = 0.139, *P* < 0.001; unweighted UniFrac: *r* = 0.063, *P* = 0.028) or suggested (Bray-Curtis: *r* = 0.048, *P* = 0.089) positive correlation with bacterial community composition in Xiloá (Fig. 4d). These results indicate that differences in trophic ecology among individuals of the same crater lake are, to some extent and dependent on the used distance metric, associated with differentiation of the gut microbiota, although correlation coefficients tended to be low in general.

**Fig. 5:**
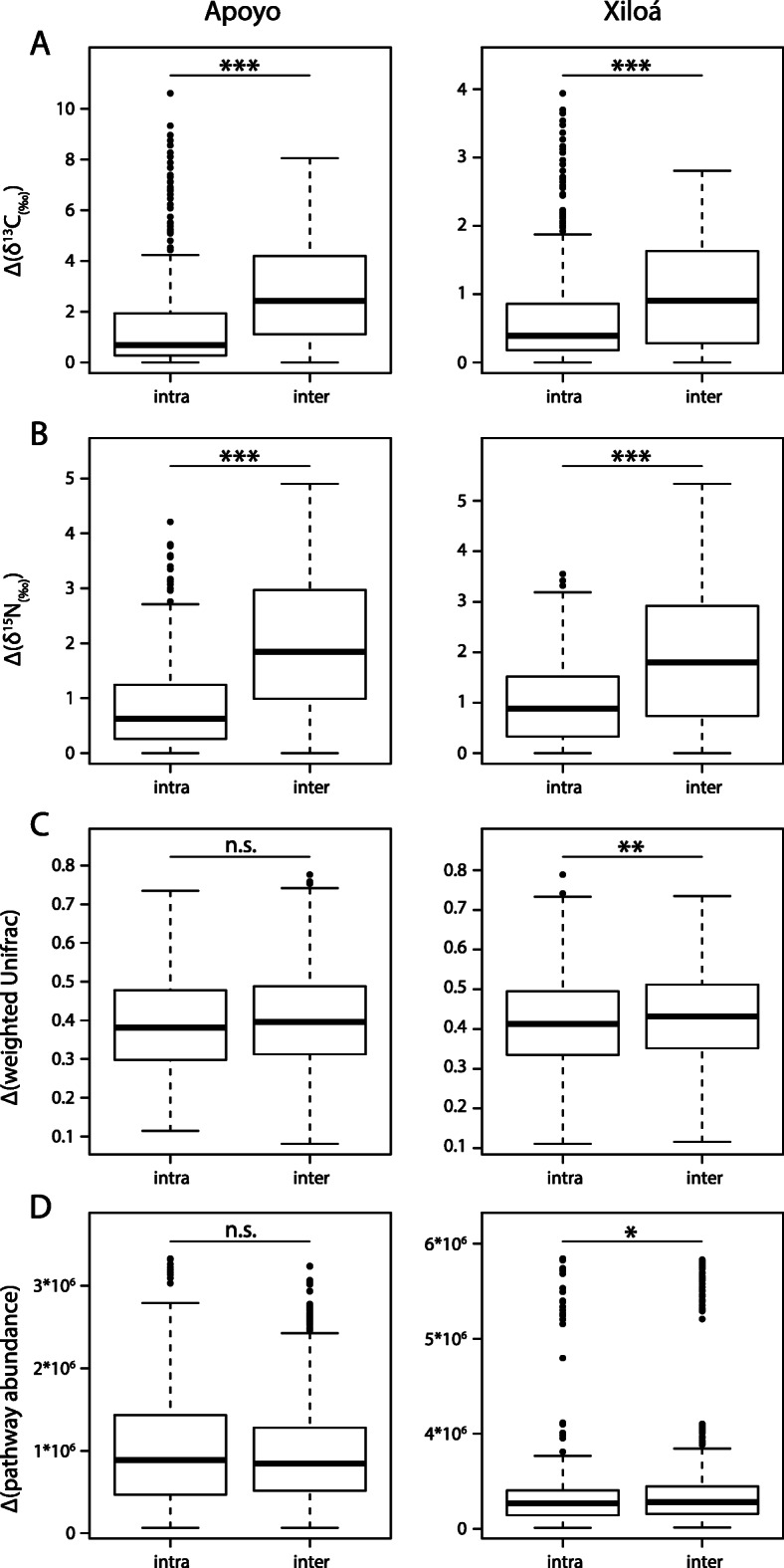
Intra- and interspecific distances in stable isotope values of carbon (**a**) and nitrogen (**b**) as well as the gut microbial community (**c**) and predicted functional bacterial metagenomes (**d**) among individuals within the two crater lakes. For carbon and nitrogen, interspecific distances are highly significantly larger than intraspecific ones. For the gut microbiota, both taxonomically and functionally, distances are much more similar and significant differences could only be seen in crater lake Xiloá (Wilcoxon rank-sum test, *P < 0.05, **P < 0.01, ***P < 0.001)

Distinguishing between intra- and interspecific comparisons of pairwise distances revealed that differentiation in the diet is more distinct among than within species for carbon and nitrogen (Wilcoxon rank-sum test, all *P* < 0.001 for both lakes; Fig. 5a, b). In contrast, taxonomic differentiation of the gut microbiota (weighted UniFrac: *P*_*Apoyo*_ = 0.069, *P*_*Xiloá*_ = 0.004; unweighted UniFrac: *P*_*Apoyo*_ = 0.031, *P*_*Xiloá*_ = 0.001; Bray-Curtis: *P*_*Apoyo*_ < 0.001, *P*_*Xiloá*_ < 0.001; see Fig. 5c for weighted UniFrac data) and the predicted functional metagenome (*P*_*Apoyo*_ = 0.824, *P*_*Xiloá*_ = 0.047; Fig. 5d) showed more similar (albeit significantly different for some metrics) levels between intra- and interspecific comparisons. These results clearly illustrate that differentiation in diet among species is not reflected by equivalent changes of the gut microbiota within crater lakes (Fig. 5).

### Parallelism and non-parallelism of the gut microbiota in crater lake Midas cichlids

Next, we investigated whether the repeated evolution of sympatric crater lake species that differ in trophic ecology is associated with parallel changes of the gut microbiota. As δ^15^N values were consistently higher in crater lake Xiloá compared to crater lake Apoyo, we performed *z*-score normalization of the data to allow comparisons across crater lakes by inferring trophic position (normalized δ^15^N) and littoral carbon usage (normalized δ^13^C) (Fig. S4; [50]).

When comparing the adaptive radiations from both crater lakes, bacterial community composition was significantly affected by the lake (adonis, *P* < 0.01 for all three metrics), but neither by trophic position (*P* > 0.05 for all three metrics) nor littoral carbon usage (*P* > 0.05 for all three metrics). The predicted functional bacterial metagenome (MetaCyc pathway abundance) was not affected by the lake (*P* = 0.191) nor by the littoral carbon usage (*P* = 0.525) but was significantly affected by trophic position (*P* = 0.005). Further, we tested which bacterial orders and inferred MetaCyc pathways were affected by trophic ecology in parallel across the two crater lakes (see the “Methods” section for more details). Without correcting for multiple testing, abundances of 21 and nine bacterial orders were affected in parallel by trophic position and littoral carbon usage values, respectively. Abundances of 15 and two MetaCyc pathways were affected in parallel by trophic position and littoral carbon usage values, respectively. This only represents 6.5% (δ^15^N) and 2.8% (δ^13^C) of the 323 bacterial orders and 0.4% (δ^13^C) of 459 MetaCyc pathways. None of these bacterial orders or MetaCyc pathways remained significant after correcting for multiple testing (FDR). Taken together, these results indicate that overall gut microbiota differentiation did not occur in parallel with divergence in Midas cichlids’ trophic ecology across the two crater lakes.

## Discussion

Numerous studies on diverse vertebrate species have convincingly demonstrated that the composition of bacterial communities of the gut is affected by diet [13, 51–54]. What remains largely unknown are gut microbiota dynamics during the host’s adaptation to novel food sources, particularly during early stages of species divergence (but see [14, 15]). To address this question, we studied trophic ecology and the gut microbiota of repeated Nicaraguan Midas cichlid crater lake radiations, a model system for rapid ecological diversification and speciation [19, 20, 29]. We asked whether the parallel evolution of trophic diversification is associated with respective changes of the gut microbiota among sympatric crater lake species (i.e., are the gut microbiotas of ecologically similar species that independently evolved in two crater lakes more similar to each other than they are to their closest relatives of the same lake?). Our results suggest that among individuals of the same crater lake differentiation in trophic ecology and the gut microbiota are, to some extent, associated, hinting at the importance of diet in shaping the gut microbiota. However, only a small proportion of gut microbiota variance is explained by differences in diet. Hence, future studies need to address the contribution of other factors to obtain a more comprehensive picture of gut microbiota dynamics in this system. Moreover, we found that interspecific variation in trophic ecology (measured as stable isotope ratios of carbon and nitrogen) is significantly higher than intraspecific variation, a pattern that was much less pronounced for the gut microbiota (Fig. 4). We did not find strong evidence for parallel changes of the gut microbiota across crater lakes, suggesting that diet affects Midas cichlids’ gut microbiotas differently in these lakes. We want to emphasize that our analyses are restricted to bacterial communities harbored within the midgut of these fish and should be interpreted in that context. Signatures of differentiation might be different if another part of the gut (e.g., hind gut) is analyzed, and future studies will need to address this question.

### Gut microbiota differentiation across lakes

Comparing bacterial communities from the water of their natural habitats with those harbored in fish guts clearly revealed that, although some bacterial taxa are shared, the gut microbiota does not merely represents the bacterial community of the natural environment. Water and gut samples were obtained at different time points and sequenced independently on different sequencing platforms (see the “Methods” section for more details). Temporal variation and technical aspects might have contributed to the observed differences in microbial communities (Fig. 2). Thus, caution should be taken when drawing conclusions about the extent to which the gut microbiota of Midas cichlids is derived from bacteria present in the water based on our data, particularly since microbial communities of lakes are known to show temporal variation (e.g., [55–57]). Future studies need to systematically investigate the temporal dynamics of microbial communities for the Nicaraguan lakes in more detail. However, the differences in microbial community composition (Fig. 2) and abundance of major bacterial phyla (Fig. 3) are strong between water and gut samples, suggesting that—given the limitations mentioned above—they might actually represent biological signals and that the gut microbiota of Midas cichlids is largely controlled by the host, as has been found for other fishes [14, 15, 58]. In accordance with a previous study on this system [17], *Proteobacteria*, *Firmicutes*, *Fusobacteria,* and *Bacteroidetes* were the dominant bacterial phyla of Midas cichlids’ gut microbiota. These bacteria are also found in many other freshwater fishes [14, 15, 59]. Albeit the bacterial diversity of environmental samples strongly differed among lakes (Fig. 3b), the gut microbiota of Midas cichlids, except for *A. citrinellus* from Lake Managua (but see [17]), showed constant levels for this measure. This provides further evidence that the diversity of bacterial species in the gut might be constrained by the host and stabilized at a given level, as predicted by the holobiont concept [60]. In *A. citrinellus* from Lake Managua, bacterial diversity was by far the lowest among all populations (Fig. 3b) and was also lower in water from Lake Managua compared to Lake Nicaragua. The city of Managua, Nicaragua’s capital with a population of more than 2 million, is located on the shore of Lake Managua and for decades, domestic and industrial waste water has been disposed into the lake [61]. As a result, concentrations of mercury and other toxic substances are extremely high in the lake and are also enriched in fishes [61, 62]. Mercury levels have been shown to be correlated with δ^15^N values [63, 64] and Midas cichlids from Lake Managua showed the highest δ^15^N values (Fig. 1), in agreement with the observation that mercury accumulates in these fishes. Further, aquatic pollutants such as heavy metals or pesticides have been shown to alter community composition and reduce the diversity of the gut microbiota in aquatic organisms (reviewed in [65]). Albeit speculative at this point, high levels of contamination might have decreased the bacterial diversity of Lake Managua as well as the gut microbiota diversity of Midas cichlids inhabiting this lake, pointing to the combined influence of host and environment in shaping the gut microbiota.

### Association between trophic ecology and gut microbiota in crater lake adaptive radiations

Midas cichlids from crater lakes Apoyo and Xiloá represent an excellent model to study the dynamics of gut microbiota changes during the early stages of ecological diversification and speciation. Species repeatedly diverged only very recently and show differentiation in trophic ecology (Fig. 1), albeit diet overlaps to varying degrees among species [24]. Therefore, we tested whether gut microbiota differentiation is associated with trophic ecology of crater lake Midas cichlids.

In general, we detected significant differences of gut bacterial community composition among sympatric species in both crater lakes, which is in contrast with previous results [17]. These discrepancies could have been the result of several differences between the two studies: (i) sample sizes were larger in our study; (ii) we included one additional species in crater lake Apoyo (*A. globosus*), which could affect the PERMANOVA test statistics; (iii) sequencing depth per individual was higher in our study after rarefaction (20,000 vs. 15,000 reads), which might increase the number of rare ASVs in our study as exemplified by the rarefaction curve (Fig. S1); and (iv) different regions of the gut might have been sampled. We found some evidence for positive correlations between the differentiation of the gut microbiota with carbon and nitrogen isotope signatures in both crater lakes (Fig. 4). However, we want to emphasize that results varied depending on distance metric (weighted UniFrac, unweighted UniFrac, Bray-Curtis dissimilarity) and statistical test (Mantel test or Pearson correlation). Overall, it appears that divergence of trophic ecology and the gut microbiota are to a certain degree associated, suggesting that adaptation to different food sources necessitated changes of the gut microbiota. But, these patterns appear not to be produced by shifts in the abundance of similar bacterial taxa as we detected no evidence for parallelism in gut microbiota changes across the two crater lakes. It should also be noted that a substantial amount of gut microbiota variation is not explained by trophic divergence. Differences in stable isotope ratios, reflecting the host’s trophic ecology, were considerably higher across species compared to within species in both crater lakes (Fig. 5a, b). In contrast, such differences were much less pronounced for the taxonomic composition of the gut microbiota and the predicted functional bacterial metagenome (albeit statistically significant in some cases; Fig. 5c, d). This could mean that occupation of novel trophic niches might be achieved without drastically changing the overall composition of the gut microbiota, both taxonomically and functionally. Rather, subtle changes in some functionally important bacterial taxa might suffice to exploit new food sources and to allow ecological and evolutionary diversification of the hosts. Alternatively, the very recent divergence of crater lake Midas cichlids might impede clear differentiation of the gut microbiota, as discussed in more detail in the following paragraph.

### Parallelism and non-parallelism of the gut microbiota in fishes

Parallel changes of the gut microbiota associated with differentiation in trophic ecology have been reported for older fish species [13], whereas other studies found no evidence for parallelism among more recently diverged populations ([14, 15], but see [16]). In the very recent Midas cichlid adaptive radiations from Apoyo and Xiloá that diverged less than 1700 and 1300 generations ago, respectively [20], parallel changes in diet led us to expect that a similar pattern could also be found in the gut microbiota. However, we did not detect evidence for parallel changes of the gut microbiota. The only exception to this was a significant association of the predicted functional metagenome with trophic position (measured as normalized δ^15^N). These results indicate that functional rather than taxonomic characteristics of the gut microbiota might be important during early stages of trophic divergence. Taken together, studies of multiple groups of fishes suggest that parallel changes of the gut microbiota might only be expected on longer time scales [13]. These observations can be explained by the fact that during early stages of divergence, species might occupy novel niches but diet, to varying degrees, still overlaps among young species or ecotypes.

In crater lake Midas cichlids, stable isotope analyses showed that species largely occupy distinct niches with varying levels of overlap among species (Fig. 1). Combining these results with previous stomach content analyses suggest that these species are generally omnivorous and mainly feed on similar food items but their relative proportions differ among species [24]. Note that the study by Elmer et al. [24] classified Midas cichlids only as benthic or limnetic; thus, variation among benthic species had not been investigated to date in this system. The results of the stable isotope analysis suggest that young crater lake species might be in the process of adapting to specialized ecological niches, but currently, they are still opportunistic generalists with a varying diet. Although Midas cichlids from the two crater lakes diverged in trophic ecology in parallel, we did not detect evidence for parallel changes of the gut microbiota. One possible explanation for the lack of microbiota parallelism is that there might be hidden variation in prey items that are not captured by stable isotope data. Stable isotope data are suitable for showing general differences in trophic ecology, but they do not provide detailed information on the exact prey items an organism feeds on. While it has been shown that prey items are largely similar between species and also across crater lakes [24], future studies that incorporate data on the gut microbiota, stable isotopes, and stomach contents are needed to investigate this possibility in more detail. Further, short-term changes of an individual’s diet are not reflected in the stable isotope signature of the muscle tissue as this represents an average of this individual’s diet over a period of approximately 3 months [66, 67]. In contrast, the composition of the gut microbiota is highly variable and changes rapidly with diet [68, 69]. Hence, the gut microbiota rather represents a snapshot of an individual’s most recently acquired food items, generating high levels of intraspecific variation. This could explain why intra- and interspecific variation of the gut microbiota is much more equal compared to stable isotope data (Fig. 5). Accordingly, high levels of intraspecific dietary variation might mask interspecific differences in trophic ecology, thereby blurring any signal of gut microbiota parallelism in recently diverged ecotypes or species. This is what we can also see in other fishes like whitefish and guppies, where the main change between ecotypes is in the relative proportion of food items [14, 15]. In contrast, benthic-limnetic species pairs of threespine stickleback show little overlap in diet [70, 71], which might explain the strong and parallel changes of the gut microbiota, despite the young age of these species [16]. Only after species sufficiently diverged to become trophic specialists that do not overlap in food items, one would expect persistent and parallel patterns of gut microbiota divergence, as seen in African cichlids or stickleback [13, 16].

## Conclusions

Here, we analyzed the gut microbiota (16S rRNA gene sequencing) as well as trophic ecology (stable isotope ratios of carbon and nitrogen) of Nicaraguan Midas cichlid fish. We found that gut microbiota composition shows host-specific signatures and strongly differs from lake water bacterial communities. Recently diverged crater lake species differ to varying degrees in trophic ecology. However, the contribution of trophic ecology to gut microbiota differentiation appears to be limited, as differences in diet did not evoke major rearrangements of gut microbial communities. While young adaptive radiations of Midas cichlids show parallel differentiation in trophic ecology across two crater lakes, such parallelism is not reflected by according shifts of the gut microbiota. This pattern seems to be common among recently diverged species of fish and could be generally explained by a lack of sufficient trophic divergence that impedes consistent and predictable shifts of the gut microbiota. This could be the case in Midas cichlids where diet shifts are associated with changes in the proportion of food items consumed rather than with occupying completely distinct trophic niches. Thus, taking into account ecological characteristics (e.g., extent of trophic divergence) as well as the evolutionary history of host species will aid in predicting when to expect parallel changes of the gut microbiota.

## Supplementary information

**Additional file 1: Figure S1.** Alpha diversity estimates at different rarefaction depths for water (black) and fish (grey) samples. The investigated sequencing depths range from 11 to 200,000 reads. At a sampling depth of 20,000 reads, a large proportion of the microbial diversity in fish guts is captured.

**Additional file 2: Figure S2.** Bacterial diversity (number of ASVs, Faith’s PD and Shannon diversity) for water and gut samples.

**Additional file 3: Figure S3.** Principal coordinate analysis of gut microbiota from crater lake Midas cichlids measured as weighted UniFrac.

**Additional file 4: Figure S4.** Trophic position and proportion of littoral carbon of crater lake Midas cichlids were inferred by performing a z-normalization of nitrogen and carbon stable isotope values.

## Data Availability

The raw sequencing datasets generated and analyzed during the current study have been deposited in the NCBI Short Read Archive under project number PRJNA615202. The R code used for downstream analyses and the underlying data files have been archived in the Dryad database: 10.5061/dryad.brv15dv6h.
