# Supplementary figures and images for: Parallel and non-parallel changes of the gut microbiota during trophic diversification in repeated young adaptive radiations of sympatric cichlid fish
Source: Microbiome. 2020 Oct 29;8:149. doi: 10.1186/s40168-020-00897-8 (PMC7597055; doi:10.1186/s40168-020-00897-8)

# Alpha rarefaction

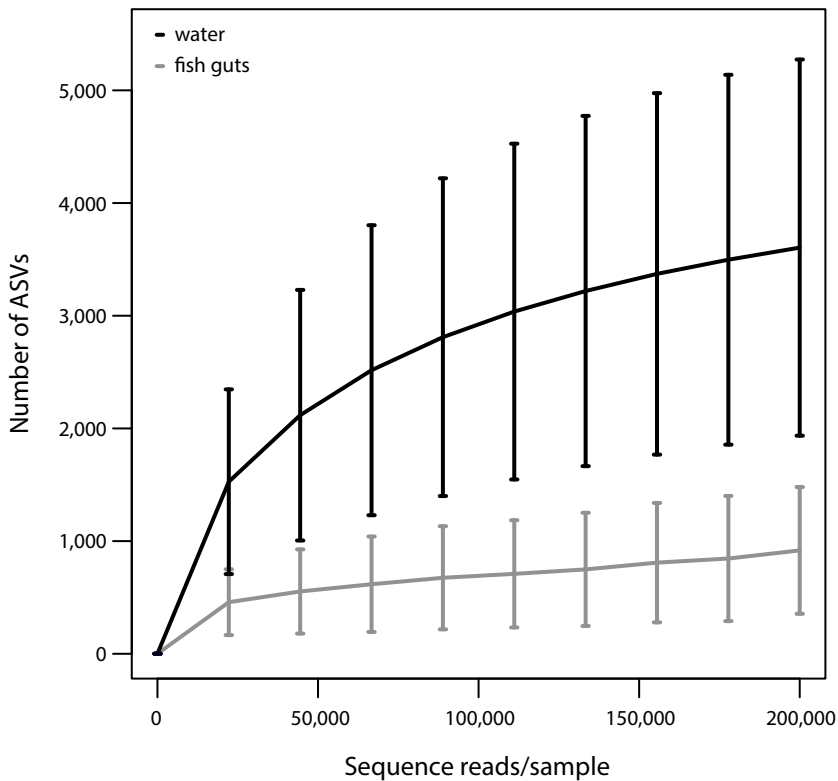

Supplement: Supplementary file 2 — Additional file 1: Figure S1. Alpha diversity estimates at different rarefaction depths for water (black) and fish (grey) samples. The investigated sequencing depths range from 11 to 200,000 reads. At a sampling depth of 20,000 reads, a large proportion of the microbial diversity in fish guts is captured. [file 40168_2020_897_MOESM1_ESM.pdf]

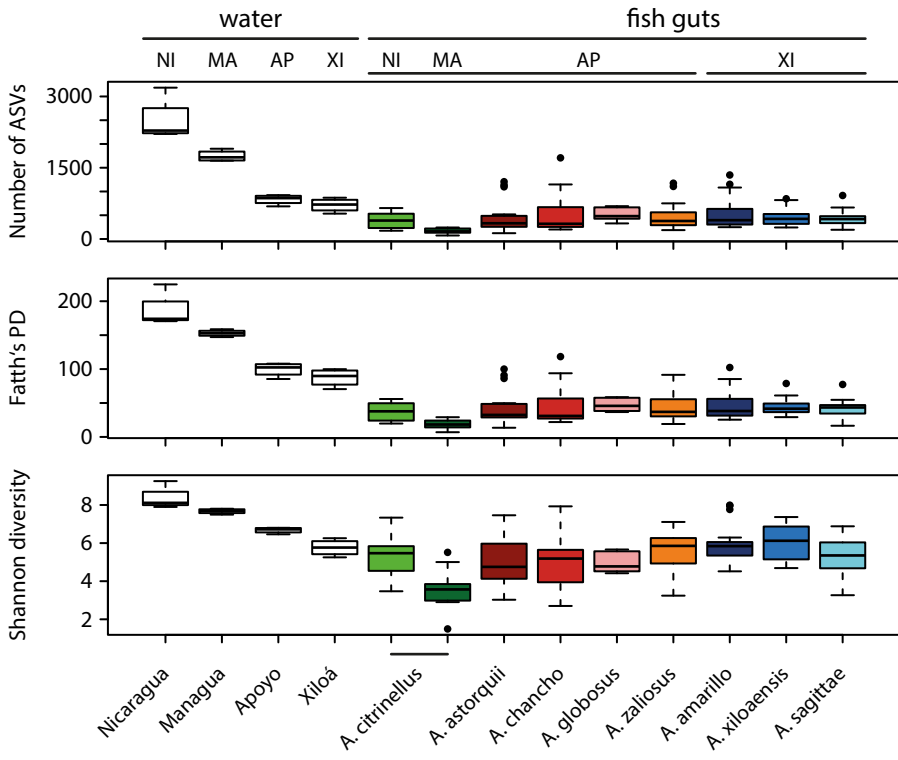

Supplement: Supplementary file 3 — Additional file 2: Figure S2. Bacterial diversity (number of ASVs, Faith’s PD and Shannon diversity) for water and gut samples. [file 40168_2020_897_MOESM2_ESM.pdf]

▲ Apoyo

■ Xiloá

*A. astorquii*

*A. amarillo*

*A. chanco*

*A. xiloaensis*

*A. globosus*

*A. sagittae*

*A. zaliosus*

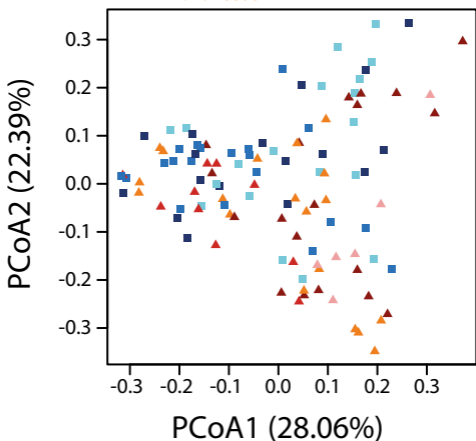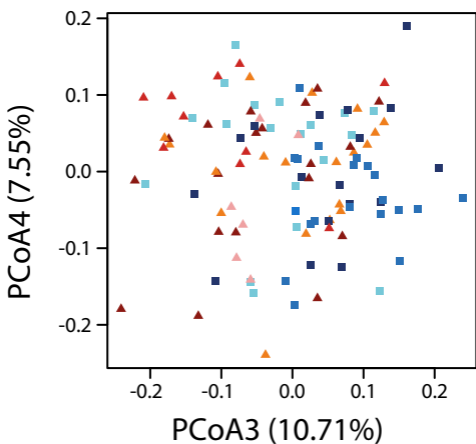

Supplement: Supplementary file 4 — Additional file 3: Figure S3. Principal coordinate analysis of gut microbiota from crater lake Midas cichlids measured as weighted UniFrac. [file 40168_2020_897_MOESM3_ESM.pdf]

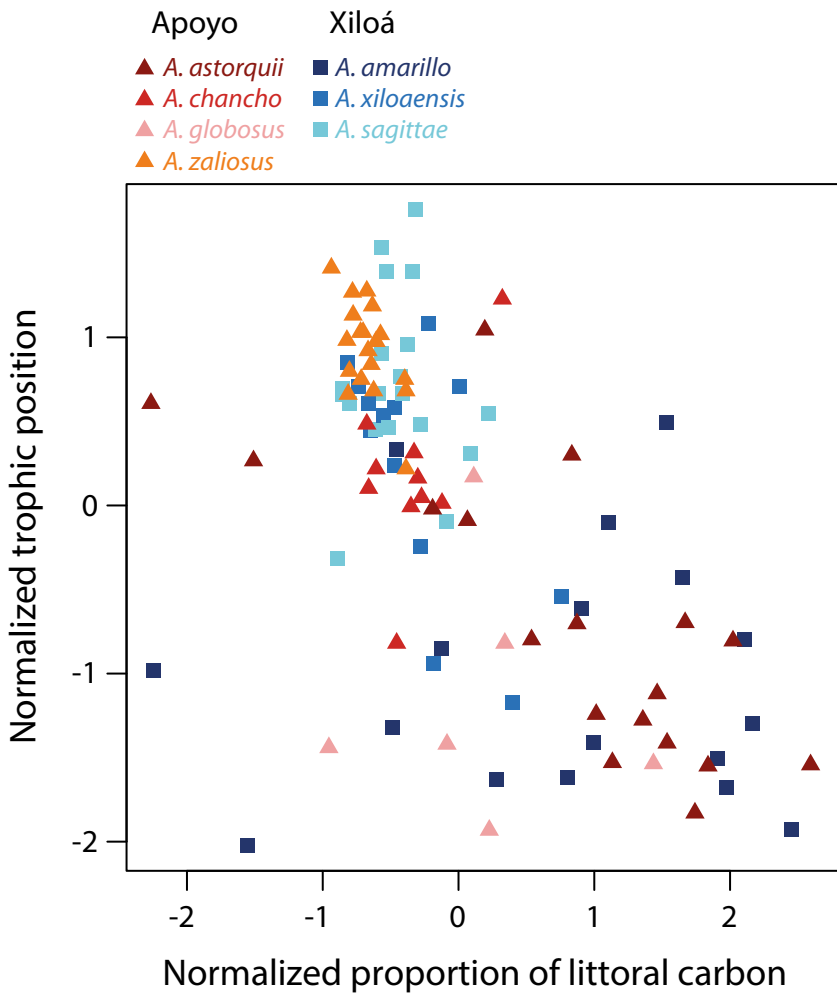

Supplement: Supplementary file 5 — Additional file 4: Figure S4. Trophic position and proportion of littoral carbon of crater lake Midas cichlids were inferred by performing a z-normalization of nitrogen and carbon stable isotope values. [file 40168_2020_897_MOESM4_ESM.pdf]
